# Strategies to deploy interventions for prevention of infection related to central venous catheter

**DOI:** 10.1186/1753-6561-5-S6-O12

**Published:** 2011-06-29

**Authors:** CA Binelli, SS Lessa, PR Daher, S Isidoro, PA Oliveira

**Affiliations:** 1 Infection Control Department, Hospital São Camilo, São Paulo, Brazil

## Introduction / objectives

Bloodstream infections related to central venous catheter are the most frequent causes of morbidity and mortality in intensive care units. Studies show that education and training of health professionals on the practice of dealing with the central venous catheter is an important tool in preventing and reducing infections related to central venous catheter. The aim of this study was to describe the experience of a Brazilian hospital in the deployment of measures to prevent infection-related central venous catheter.

## Methods

This is an experience report performed in a midsize charity institution in the city of São Paulo, Brazil, conducted from February 2009. Health institutions seek constantly to improve their practices. Based on that principle, the team was redefined; and an electronic check-list was deployed containing information about the care during insertion. Also manipulation and dressing of central venous catheter were performed with daily fulfilment by the medical and nursing teams, besides a training to all professionals involved with the care of catheters. Data collection, critical analysis and presentation of monthly results were also performed to senior managers and health professionals.

## Results

After the project was done, a percentage of compliance was observed at 87% for completing the checklist, and a reduction of blood stream infections related to central venous catheter from 1.3 per 1.000 catheter/day to 0.5 infections related to central venous catheter per 1.000 catheter/day in 2009.

## Conclusion

Therefore, it was possible to conclude that the deployment of interventions contributed to the reduction of bloodstream infection fees related to central venous catheter.

## Disclosure of interest

None declared.

